# Riddelline from *Tamarix articulate* as a potential anti-bacterial lead compound for novel antibiotics discovery: A comprehensive computational and toxicological studies

**DOI:** 10.1371/journal.pone.0310319

**Published:** 2024-11-14

**Authors:** Abdullah M. Alnuqaydan

**Affiliations:** Department of Basic Health Sciences, College of Applied Medical Sciences, Qassim University, Buraydah, Saudi Arabia; Abasyn University, Peshawar, Pakistan, PAKISTAN

## Abstract

*Tamarix articulate* from the Tamaricaece family is a halophytic plant. This plant is commonly called Athal or *Tamarix* in different Arabic and Asian countries. Due to the high load of polyphenolic phytochemicals, the plant has been used as a therapeutic option against several diseases for decades. The plant is an anti-inflammatory, anti-bacterial, anti-viral, anti-cancer, anti-oxidant, and anti-inflammatory. In this work, the 222 phytochemical compounds of *T*. *articulate* from our previous study are used in different bioinformatic and biophysics techniques to explore their biological potency against different anti-bacterial, anti-cancer and anti-viral targets. By doing so, it was found that Riddelline ranked as the best binding molecule of biological macromolecules selected herein in particular the bacterial targets. The binding energy value of the compound for the KdsA enzyme was -14.64 kcal/mol, KdsB (-13.09 kcal/mol), MurC (-13.67 kcal/mol), MurD (-13.54 kcal/mol), MurF (-14.20 kcal/mol), Polo-like kinase 1 (Plk1) (-12.34 kcal/mol), Bcl-2 protein (-13.39 kcal/mol), SARS-CoV-2 main protease enzyme (-12.67 kcal/mol), and Human T cell leukemia virus protease (-13.67 kcal/mol). The mean Rg value of KdsA-Riddelline complex and KdsA-FPE complex is 32.67 Å, and average RMSD of KdsA-Riddelline complex and KdsA-FPE complex is 2.31 Å, respectively. The binding energy complexes was found to be dominated by van der Waals (-71.98 kcal/mol for KdsA-Riddelline complex and -65.09 kcal/mol for KdsA-FPE complex). The lead compound was also unveiled to show favorable druglike properties and pharmacokinetics. Together, the data suggest the good anti-bacterial activities of the *T*. *articulate* phytochemicals and thus can be subjected to experimental in vitro and in vivo investigations.

## Introduction

The experimental drug development of novel drugs is a rocky process and often faces a variety of challenges [1]. As a result of the hurdles, few drug candidates transformed from hit/lead compounds to commercial products due to many reasons including off-target effects, low solubility, less stability, physicochemical properties, and poor binding affinity [2]. The process is additionally complicated by the high cost and extended period [3]. Considering these limitations, it is, therefore, important to optimize each step of the drug discovery to maximize success chances [4]. The use of computer power is now an integral component of drug discovery to fast track novel drugs identification and commercialization [5]. This process is known as computer aided drug discovery. By computer aided drug discovery, several successful drugs have been developed. For example, oseltamivir, dorzolamide, captopril, saquinavir, zanamivir, and aliskiren are some drugs in clinical trials or approved for use [6].

For millennia, aromatic and medicinal plants have been a source of remedies for variety of diseases [7]. These plant-based medications were initially presented as inhalations, infusions, teas, powders, tinctures, poultices, decoctions, etc. [8]. The scientific investigation of medicinal plants for years aimed to disclose novel drugs [9]. This can be exemplified by several examples such as Paclitaxel which is recovered from *Taxus brevifolia* and used for treating breast, ovarian and lung cancers [10]. A Silymarin compound from *Silybum marianum* plant is employed for treating hepatic disorders [11]. Similarly, Cocaine is used for stimulating perspiration and salivation [12]. Plants based drugs are cost-effective compared to microbial fermentation and animal cell cultures [13]. The drugs from plants are structurally diverse and can served as parent compounds for additional derivatives development [14]. *Tamarix articulate* (known as *T*. *articulata*) is a halophytic medicinal plant from Tamaricaece family and is commonly known as Athal or Tamarisk in Arabic countries [15]. The plant is considered a good source of polyphenolic phytochemicals and since ancient times, the plant either alone or in combination with other different plant extracts has been used against a variety of different ailments [16–18]. Several studies reported the use of *T*. *articulate* in several therapeutic applications due to its wide spectrum and diverse phytochemicals [19]. The plant is frequently reported to possess the potential of anti-inflammatory, anti-bacterial, anti-viral, anti-cancer, anti-oxidant, and anti-inflammatory [20–23]. Considering the wide range of pharmacological properties of *T*. *articulate*, there is an open window for extensive research on the plant for getting access to the potent biological active phytochemicals that be utilized in future therapeutic research [16,24].

Regarding this opportunity, in the current research work, several bioinformatic and biophysics techniques are applied using 222 phytochemical compounds from our previous work and evaluate their potential as anti-bacterial, anti-viral, and anti-cancer [24]. The important techniques used in the work include structure based virtual screening [25], all atoms molecular dynamics simulation [26], binding free energies estimation by Molecular Mechanics/Poisson-Boltzmann Surface Area (MM/PBSA), and Molecular Mechanics/Generalized Born Surface Area (MM/GBSA) [27], binding entropy energy investigation [28], WaterSwap [29] and absorption, distribution, metabolism, excretion, and toxicity (ADMET) properties analysis [30–32]. All these analyses ensure the selection of a potent lead from the list of *T*. *articulate* phytochemicals and cross-validate the findings. The virtual screening results often are victim of false positive data, that was validated by long term molecular dynamics simulation. This analysis helps not only validate the docking results by deciphering the long simulation time conformation stability of the screened compound against the high affinity binding receptor but also discloses the binding mechanism of the ligand and its interactions pattern. These atomic level findings can increase our knowledge of optimizing the ligand structure to achieve greater biological activity and provide a platform for additional compound derivates with high anti-spectrum. Further, different binding free energies were predicted which together aid in easing drug development stages. The ADMET properties analysis furthermore points about the selected compound lead pharmacokinetic properties in real cell environment. Together, the findings of the study will expedite the search for novel leads and speed up drug discovery process against different diseases.

## Materials and methods

**Fig 1:**
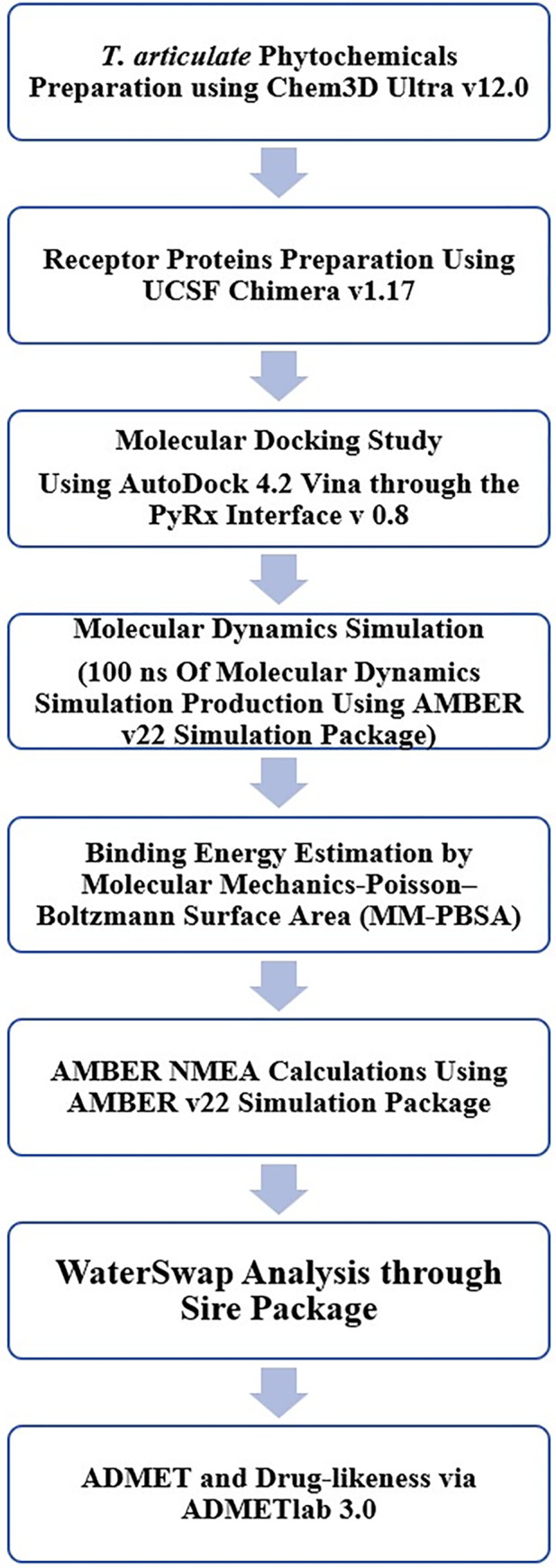
Schematic flow of the research methodology applied in the study for achieving the objectives.

The complete flow of the methods applied to achieve the study objectives is provided in **Fig 1.**

### *T*. *articulate* Phytochemicals preparation

The phytochemicals of *T*. *articulate* (which were total of 222 compounds as given in **S1 Table**) were drawn in ChemDraw v12.0 software [33]. The compounds were then fetched into Chem3D v12.0 to energy optimize the compounds structure using MM2 force field [33,34]. Afterward, the compounds were saved as.pdb to be utilized in docking calculations.

### Receptor proteins preparation

The receptors used in this work were selected from different infectious pathogens and cancer. The targets used were; KdsA (PDB code; 1PHQ) [35], KdsB (PDB code;3POL) [36], MurC (PDB code;5VVW) [37], MurD (PDB code;5A5F) [38], MurF (PDB code;4QDI) [39], Polo-like kinase 1 (Plk1) (PDB code;8CRC) [40], Bcl-2 protein (PDB code;2O2F) [41], SARS-CoV-2 Main Protease Enzyme (PDB code;7TUU) [42], and Human T Cell Leukemia Virus Protease (PDB code;2B7F) [43]. These receptors were fetched to UCSF Chimera v1.17 for energy minimization process [44]. During this event, steepest descent (1500 rounds) and conjugate gradient (1500 rounds) algorithms were applied to get structure optimized receptors. Both the energy minimization algorithms were run for 3000 rounds with a step size of 0.02 Å.

### Molecular docking study

The AutoDock 4.2 Vina through the PyRx interface v 0.8 was employed for molecular docking studies [45,46]. The docking was carried out using Lamarckian Genetic Algorithm (LGA) with different parameters such as crossing rate of 0.8, evaluations number of 250,000 and 27000 generations. The docking was performed at the active region of the enzymes, which was based on literature review. The validation of the docking procedure was done to ensure that the study produce intermolecular docked pose and interactions as that of a known crystal receptor-ligand complex. The docked compounds were listed in descending order; the most negative binding energy compounds (in kcal/mol) were found the best docked molecules to the receptor biomolecules [47]. The poseview of the compounds/receptors was visualized through Discovery Studio v2024 [48].

### Molecular dynamics simulation

100 ns of molecular dynamics simulation production run was conducted for selected top complex using AMBER v22 simulation package [49]. A control complex was also simulated for comparative analysis to draw molecular insights into binding mode and interactions about the opted lead molecule [26]. Information about the complex topology was extracted using an antechamber program, where FF19SB was applied to the receptor [50,51]. The GAFF2 forced field parameterized the lead and controlled organic compounds [52]. The complex was then placed into OPC box (dimensions 12 Å) filled with periodic water molecules. 9 Na+ and 15 Cl- were added to the OPC water box to neutralize the complex [53]. The total concentration achieved for neutralization is 0.15 molar. Both the lead and control molecule complex were energy minimized; steepest descent and conjugate gradient. Both the energy minimization algorithms were applied for a total of 6000 rounds. The complexes were then equilibrated through NVT (Constant temperature, constant volume) and NPT (Constant temperature, constant pressure) ensembles [54,55]. In the equilibrium protocol, pressure of 1.0 bar, temperature of 300 K and equilibrium time of 500 ps. The long-range interactions were estimated by using multiple time stepping particle mesh Ewald technique with 1.2 maximum grid spacing. During production run, the temperature and hydrogen bonds were kept constant through Langevin and SHAKE algorithms [56,57]. The simulation trajectories were produced through 2 femtoseconds time intervals. The CPPTRAJ AMBER module was utilized for structure stability analysis and run along the simulation frames [58]. The CPPTRAJ was run for radius of gyration (Rg), root mean square fluctuation (RMSF), and root mean square fluctuation (RMSF) [54,59,60]. All these analyses were done based on carbon alpha atoms.

### Binding energy estimation by Molecular Mechanics-Poisson–Boltzmann Surface Area (MM-PBSA)

The lead and control complex were further used in binding free energy estimation, which is approximated based on Molecular Mechanics-Poisson–Boltzmann Surface Area (MM-PBSA) technique [61,62]. This method considered the intermolecular interactions between the receptor enzyme and lead/control in solvation and gas states. All different binding energies were estimated in both phases except for entropy energy. The energies include non-bonded van der Waals and electrostatic interactions energy. The other energies involve torsion, bond and angle energies as molecular mechanics energy. The solvation energy part of the technique comprises polar and non-polar energies which are computed through an implicit solvation model. The equation used for MMPBSA is;

ΔG=ΔGMM+ΔGsolvation

The procedure was applied to a total of 10,000 frames, which were picked along the simulation trajectories and from equal time intervals.

### AMBER NMEA calculations

The AMBER Normal Mode Entropy Analysis (NMEA) method was further applied on selected frames of simulation trajectories for calculation of rotational, translational and vibrational entropies [28]. The NMEA is based on optimized structures Hessian matrix and flexible protein states around equilibrium position. The NMEA analysis was not considered during MMPBSA analysis due to the use of high computational power, therefore, used separately and applied to only 10 simulation frames.

### WaterSwap analysis

The MMPBSA binding free energies were reconfirmed through another method called WaterSwap, which is implemented in the Sire package allowing estimation of intermolecular absolute binding free energies from condensed phase simulations [29,63]. The reaction coordinate of this technique allows swapping of the bounded lead/control to the receptor of equal water molecules volume. This permits consideration of water molecules role in bridging protein residues atoms and ligand atoms interactions [29]. The WaterSwap costs around 1–4 days and takes an average of Bennetts Acceptance Ratio (BAR), thermodynamic integration (TI), and free energy perturbation (FEP). The WaterSwap was run for 1000 iterations and an average of < 1 kcal/mol among the three algorithms is considered significant [64].

### ADMET and drug-likeness

The ADMET properties of the selected lead/control were investigated through the ADMETlab 3.0 server [65]. Different properties of the compounds were elucidated including physicochemical, drug-likeness, solubility, lipophilicity, pharmacokinetics, and medicinal chemistry and toxicity [66,67].

## Results and discussion

### Virtual screening of the *T*. *articulate* phytochemicals

**Fig 2:**
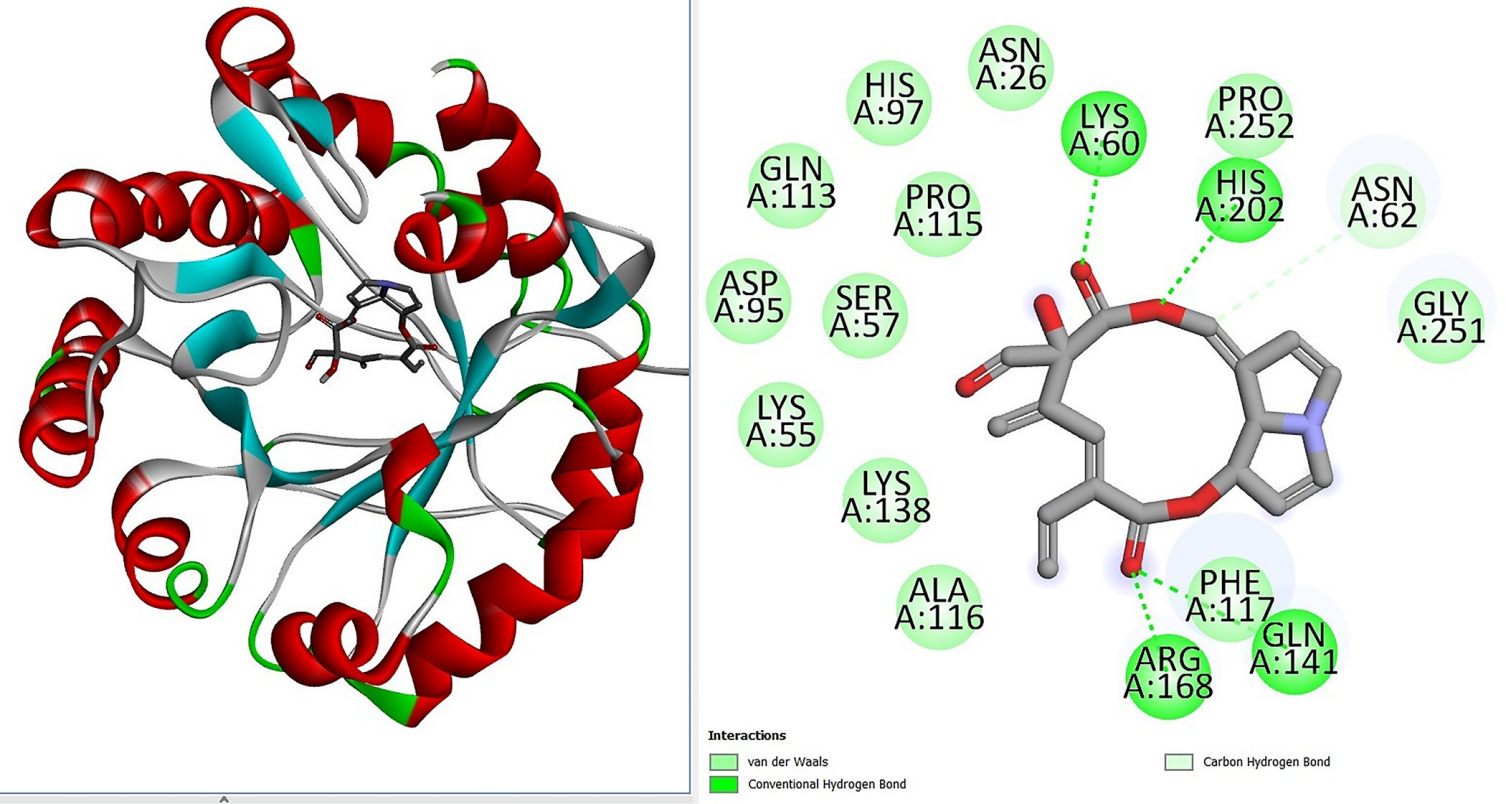
Atomic level interactions between the KdsA enzyme and shortlisted best binding ruddekkube compound. The KdsA enzyme is colored as per secondary structure elements and is in cartoon. The compound is set in stick presentation.

**Fig 3:**
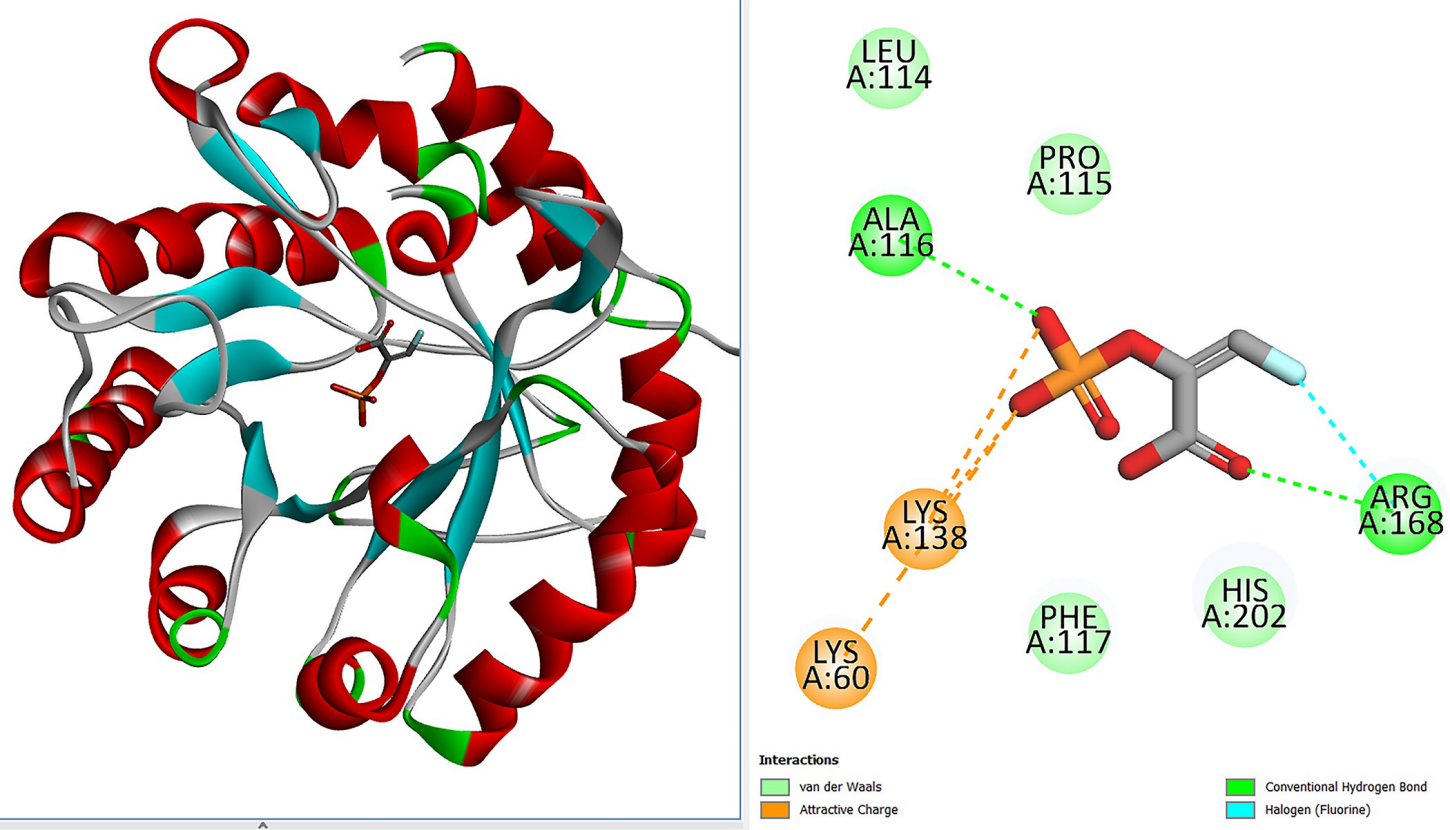
Deep binding mode of the FPE control with the KdsA enzyme. The enzyme secondary structure elements are shown in different colors and presented in cartoon type. The FPE control is given in stick. The chemical interactions between the enzyme and control are also given in the figure.

Virtual screening is an *in-silico* method employed to search small drug molecule libraries against any given macromolecules [25]. This was done to uncover compounds that showed best binding interactions and binding mode within the active pocket of the macromolecule. The structure based virtual screening is considered as a critical phase in modern drug discovery because its efficiency, shortlisting promising classes of inhibitors and reducing overall drug discovery protocol [68]. Herein, the structure based virtual screening approach was utilized to screen *T*. *articulate* phytochemicals against variety of different druggable targets from bacteria, viruses and cancer. The bacterial drug targets used were; KdsA, KdsB, MurC, MurD, and MurF ligase enzymes. The KdsA enzyme is 3-deoxy-D-*manno*-octulosonate 8-phosphate (KDO-8-P) synthase which is involved in the biosynthesis of in lipopolysaccharide [61]. The KdsB is 3-deoxy-manno-octulosonate cytidylyltransferase enzyme which performs activation of 3-Deoxy-D-mano-oct-2-ulosonic acid during Gram-negative bacteria lipopolysaccharide synthesis [36]. The Mur ligases are vital in bacterial peptidoglycan biosynthesis [37]. The MurC, MurD, and MurF are UDP-N-acetylmuramate-L-alanine ligase, UDP-*N*-acetylmuramoyl-L-alanine—D-glutamate ligase and UDP-N-acetylmuramoyl-tripeptide-D-alanyl-D-alanine ligase, respectively. All the Mur ligases share same topology and have N-terminal, central, and C-terminal domains [69]. The Serine/threonine-protein kinase Plk1 is a prot-oncogene and is highly expressed in tumor cells [70]. The Bcl-2 protein dysregulation can halt tumor progression and thus the protein is druggable and is a target for inhibitors [71]. The SARS-CoV-2 main protease enzyme plays a vital role in virus replication and is an ideal drug target [42]. The human T-cell leukemia virus type 1 (HTLV-1) protease was also selected as anti-virus drug target that’s necessary for virus maturation [72]. The 222 phytochemicals of *T*. *articulate* were screened against all the selected targets and docking calculations were repeated in triplicates. By this analysis, variety of compounds were shortlisted that showed good binding affinity for all the targets used. Comparative analysis revealed majority of the top 10 compounds showed stable binding with anti-bacterial targets **(Table 1).** In particular, the top lead molecule named Riddelline was opted as best binding molecule of the targets considered in this work. The binding energy value of the compound for KdsA (-14.64 kcal/mol), KdsB (-13.09 kcal/mol), MurC (-13.67 kcal/mol), MurD (-13.54 kcal/mol), MurF (-14.20 kcal/mol), Polo-like kinase 1 (Plk1) (-12.34 kcal/mol), Bcl-2 protein (-13.39 kcal/mol), SARS-CoV-2 Main Protease Enzyme (-12.67 kcal/mol), and Human T Cell Leukemia Virus Protease (-13.67 kcal/mol). Among the antibacterial targets, Riddelline formed highly stable interactions with the KdsA enzyme. This complex was processed for indepth binding mode and chemical interactions. Chemically, Riddelline is (3E,9Z)-6-hydroxy-5-methylene-2,7-dioxo-3-vinyl-2,5,6,7,9a1,14a-hexahydro-[1,6]dioxacyclododecino[2,3,4-gh]pyrrolizine-6-carbaldehyde. The compound was observed to accommodate itself deep inside the central cavity of the KdsA enzyme. From an interaction network point of view, the compound is engaged by four hydrogen bonds from the cavity wall. The acetic acid chemical moiety of the compound formed two hydrogen bonds with residues from opposite sides of the cavity. The bond distance of the compound with Lys60, His202, Arg168 and Gln141 is 2.54 Å, 2.69 Å, 3.01 Å and 1.84 Å, respectively. Besides this, the compound can be seen engaged in several weak van der Waals forces of the enzyme residues. These residues are; Asn26, His97, Pro115, Gln113, Ser57, Asp95, Lys55, Lys138, Ala116, Phe117, Gly251, Asn62, and Pro252 **(Fig 2)**. The bond distance of majority van der Waals interactions is within 3 Å. This suggests that both hydrogen bonds and van der Waals interactions are vital in the docked stability of the Riddelline with the KdsA enzyme. The control FPE occupied the same pocket and produced a binding affinity score of -9.20 kcal/mol. The interaction pattern involves two hydrogen bonds (with residues Ala116 and Arg168), four van der Waals (Leu114, Pro115, Phe117 and His202), and two attraction charges (Lys60 and Lys138) **(Fig 3)**.

**Table 1:** Virtual screening of the *T*. *articulate* compounds against different drug targets from pathogenic bacteria, viruses and cancer. PDB ID of each target is also given. The binding energy is reported in kcal/mol.

Compound	KdsA (1PHQ)	KdsB (3POL)	MurC (5VVW)	MurD (5A5F)	MurF (4QDI)	Polo-like kinase 1 (Plk1) (8CRC)	Bcl-2 protein (2O2F)	SARS-CoV-2 main protease enzyme (7TUU)	Human T cell leukemia virus protease (2B7F)
Riddelline	-14.64	-13.09	-13.67	-13.54	-14.20	-12.34	-13.39	-12.67	-13.67
Oxymatrine	-9.21	-8.01	-9.84	-9.38	-9.88	-10.55	-12.19	-13.64	-12.06
Bursehernin	-8.14	-7.39	-8.67	-9.34	-9.31	-11.06	-11.79	-12.09	-13.08
Casimiroedine	-13.85	-13.67	-13.78	-13.04	-13.93	-12.33	-12.04	-11.09	-10.54
Resveratrol	-12.03	-11.38	-12.36	-12.69	-11.87	-12.69	-12.67	-10.29	-11.97
Absinthin	-13.04	-12.38	-13.6	-12.66	-13.61	-10.33	-9.68	-8.86	-9.38
Visnadin	-12.38	-12.75	-12.39	-12.48	-12.44	-9.23	-10.29	-9.66	-10.39
Plicamine	-11.06	-12.39	-12.73	-11.62	-11.87	-11.64	-10.98	-10.69	-11.39
Verbascoside	-13.95	-11.01	-12.09	-12.09	-12.38	-10.36	-11.29	-8.36	-10.16
Stylopine	-12.48	-10.68	-11.79	-11.69	-11.84	-11.35	-11.98	-10.87	-10.76

### Molecular dynamics simulation

**Fig 4:**
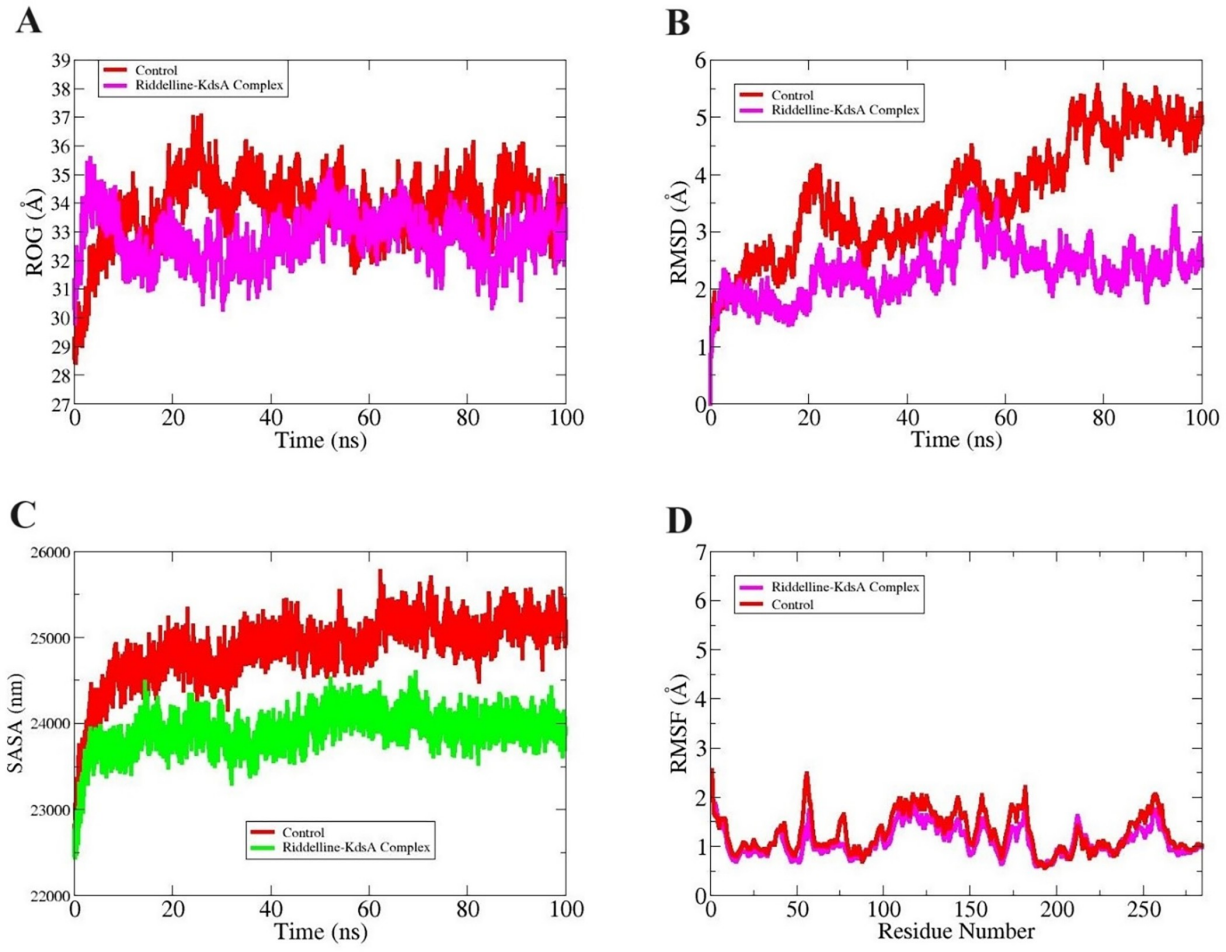
Results from molecular dynamics simulation. RoG (A), RMSD (B), SASA (C), and RMSF (D).

Proteins as macromolecules in living systems play several vital functions [73]. These functions are correlated to protein structure and investigating protein atomic structure as a function of time is important. Computational and mathematical modeling approaches can be used in this regard to evaluate the protein structure dynamics that can yield many key findings [74]. Today, molecular dynamics simulations are a powerful method to unveil information about protein structure. The simulation technique provides insights into the microscopic level of information and translates it into macroscopic properties. From a drug discovery perspective, the molecular dynamics simulation is key for unveiling the stability of organic compounds or peptides at the docked site of the KdsA enzyme. The first analysis done during molecular dynamics simulation is radius of gyration (Rg) which illustrates structure compactness along the simulation time [59]. Both the KdsA-Riddelline complex and KdsA-FPE complex were seen to show minor fluctuation along the simulation time but were observed to converge towards the end. These minor changes are the reason for high percentage of loops in the KdsA enzyme structure particularly at the N-terminal however, the secondary element changes are not affecting intermolecular binding and interactions. The mean Rg value of KdsA-Riddelline complex and KdsA-FPE complex is 32.67 Å and 35.01 Å, respectively **(Fig 4A)**. The root mean square deviation (RMSD) defines the distance changes of superimposed protein atoms at different simulation times [60]. The average RMSD of KdsA-Riddelline complex and KdsA-FPE complex is 2.31 Å and 3.42 Å, respectively **(Fig 4B)**. The control complex was seen with increasing RMSD reaching a maximum of 5 Å. On the other hand, the lead complex’s maximum RMSD achieved is 3 Å and afterward, the KdsA-Riddelline complex reaches stable equilibrium. The higher RMSD of the control complex is due to continuous conformation changes of the control molecule pressing the KdsA loops to be more flexible compared to the lead complex. The next analysis done was solvent accessible surface area (SASA) [75]. The SASA plays a significant role in structure-function of biological macromolecules. The water molecules at the surface of proteins can affect the water molecules interactions at the active site with the ligand. The SASA value of KdsA-Riddelline complex and KdsA-FPE complex is 23541 nm^2^ and 24951 nm^2^, respectively **(Fig 4C)**. The last analysis done was root mean square fluctuation (RMSF) which tells about the residues level fluctuation throughout simulation time [54]. The residue fluctuations of the lead complex are smaller than that of the control complex. The mean RMSF value of KdsA-Riddelline complex and KdsA-FPE complex are 1.21 Å and 1.42 Å, respectively **(Fig 4D)**. All the simulation results reported both the complexes are highly stable in terms of the docked compound and interactions network.

### Estimation of MMPBSA binding free energies

The MMPBSA and its counterpart MMGBSA is a powerful computational method for estimating binding free energies of protein-ligand complexes [76]. The energies are estimated by continuum solvation models and molecular mechanics principals. Both the MMGBSA and MMPBSA calculates free energies difference between bound and unbound states of either two solvation binding conformation of the same molecule or two different solvated molecules [77]. These methods are regarded as more robust than the docking algorithms which give only a single intermolecular binding conformation. The MMPBSA and MMGBSA findings are more comparable to experimental results than the docking predictions. The calculated binding free energies of the selected lead Riddelline and control FPE are tabulated in **Table 2.** The data indicates that both docked complexes reported significant net binding energy, which is -79.81 kcal/mol for KdsA-Riddelline complex and -65.42 kcal/mol for KdsA-FPE complex. As per the values, the former complex can be considered more stable in terms of binding energy. The net value of binding energy is dominated by van der Waals in both complexes (-71.98 kcal/mol for KdsA-Riddelline complex and -65.09 kcal/mol for KdsA-FPE complex). The electrostatic energy of the complexes though not significant as that of van der Waals but still played a vital role in overall complexes docked stability. The net electrostatic energy of KdsA-Riddelline complex and KdsA-FPE complex is -22.09 kcal/mol and -14.28 kcal/mol, respectively. The solvation energy in both MMGBSA and MMPBSA was found to contribute negatively towards complexes docked stability and opposed formation of complex formation. The MMPBSA results trend was found the same in MMGBSA. The MMGBSA and MMPBSA methods are not routinely used in computational drug design studies to identify potential lead molecules against variety of druggable targets [78–80].

**Table 2:** Free energies generated during complex formation between the KdsA enzyme and Riddelline/FPE control. The energies were measured in term of kcal/mol.

Energy Parameter	KdsA-Riddelline Complex	KdsA-FPE Complex
**MMPBSA**		
Van der Waals	-71.98	-65.09
Electrostatic	-22.09	-14.28
Gas phase	-94.07	-79.37
Solvation energy	14.26	13.95
Net energy	-79.81	-65.42
**MMGBSA**		
Van der Waals	-71.98	-65.09
Electrostatic	-22.09	-14.28
Gas phase	-94.07	-79.37
Solvation energy	12.88	11.56
Net energy	-81.19	-67.81

### NMEA calculations

The NMEA involves harmonic oscillator entropies, translation, and rotational degree of solute freedom. The configurational entropy calculation in MMPBSA and MMGBSA was omitted to its high computational cost and thus was performed through NMEA method considering only five frames picked from along the simulation time. Details of the entropy calculations are provided in **Table 3**. As can be seen in the **Table** 3 and can be concluded that both the complexes have less random energy suggesting the ligands are enjoying time in the receptor active pocket which ultimately leads to stable complexes formation. The lead Riddelline complex with the KdsA enzyme possesses less energy than the control FPE, demonstrating the more stable nature of the selected compound. The net binding entropy energy of KdsA-Riddelline complex and KdsA-FPE complex was found as -1.29 kcal/mol and 4.09 kcal/mol, respectively.

**Table 3:** Entropy energies of the complexes in kcal/mol.

Complex	Translational	Vibrational	Rotational	ΔS Total
**KdsA-Riddelline Complex**	11.58	14.69	1231.55	-1.29
**KdsA-FPE Complex**	12.35	17.99	1447.94	4.09

### WaterSwap calculations

**Fig 5:**
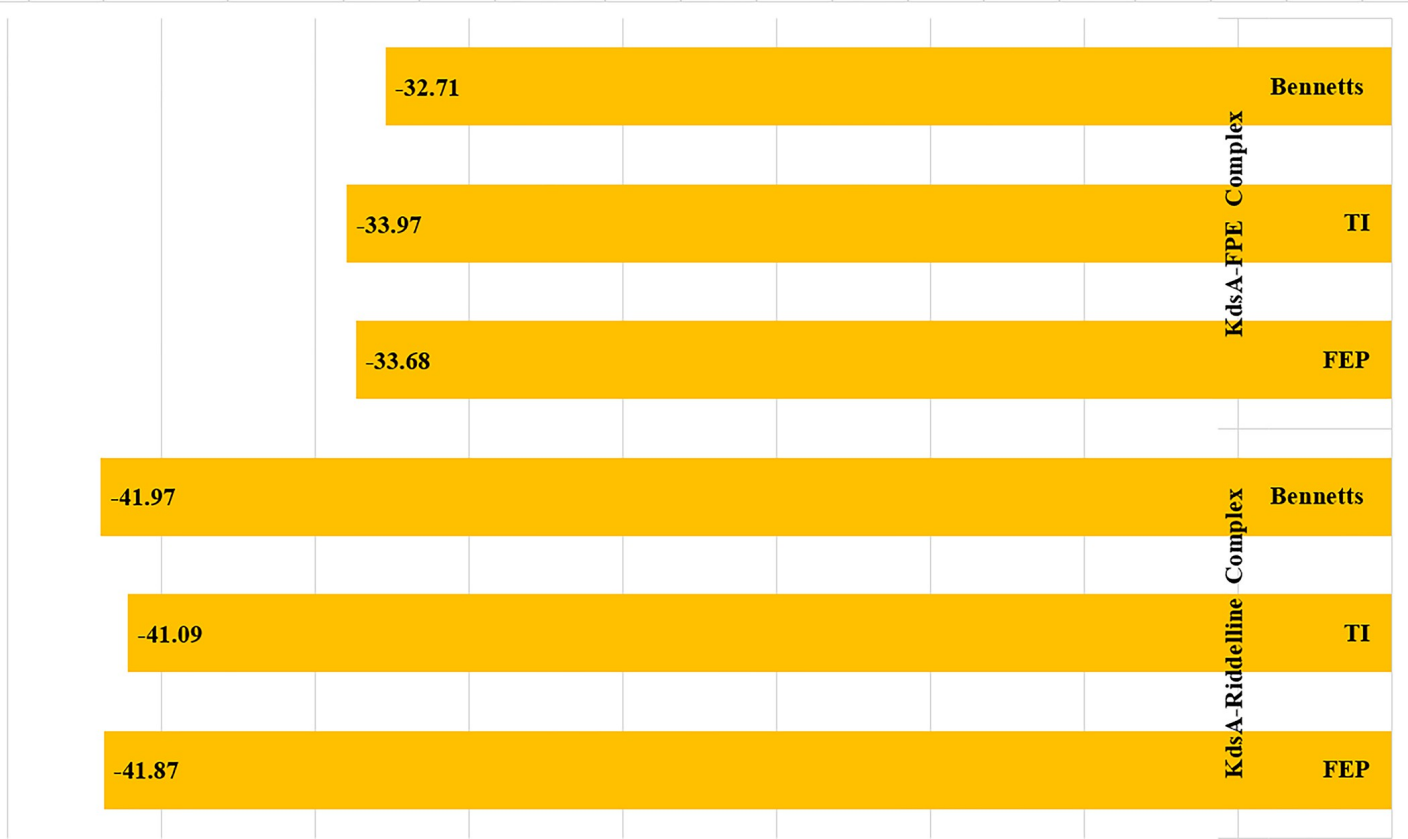
Binding free energy of algorithms used in the WaterSwap in kcal/mol.

The double decoupling cavitation and large value problems of common binding free energy methods such as that of MMPBSA and MMGBSA can be overcome by using WaterSwap method [63]. This method is considered for predicting absolute binding free energy of complexes by considering the details of water-ligand, water-protein, and protein-water-ligand interactions which is possibly due the use of an explicit water model. The WaterSwap works on a single simulation principle to swap same shape/volume of water molecules bulk to ligand bound to the receptor protein. The WaterSwap highlights the water molecules that play significant role in bridging ligands to the protein residues at the active pocket. The WaterSwap employs three algorithms for predicting complexes binding free energy. These are TI, FEP, and Bennett’s as given in **Fig 5**. The TI, FEP and Bennett’s energy values for KdsA-Riddelline complex are -41.09 kcal/mol, -41.87 kcal/mol and -41.97 kcal/mol, respectively. The WaterSwap values for KdsA-FPE complex is -33.68 kcal/mol (FEP), -33.97 kcal/mol (TI) and -32.71 kcal/mol (Bennett’s). By findings, the lead molecule is more robust in interactions with the KdsA enzyme.

### ADMET properties analysis

Details about Riddellin ADMET properties are given in **S1 File**. ADMET properties prediction is vital in selecting potential leads for drug development. This allows the selection of drug candidates of high quality at appropriate therapeutic doses. The Riddellin molecular weight is 341.09 Dalton, volume of 334.225, density of 1.021, number of hydrogen bond acceptor is 7, number of hydrogen bond donor is 1, number of rotatable bonds is 3, number of rings is 3, the size of maximum ring is 16, flexibility of the compound is 0.083, TPSA value if 93.14, LogS is -3.733, LogP is 1.551 and LogD is 1.542. The synthetic accessibility score of the compound is 5.927 which indicates the selected Riddellin can easily be synthesized for in vitro and in vivo analyses [81]. The compound was further classified as druglike molecule according to Lipinski’s rule of five [82], Pfizer’s rule, GSK rule, and Golden triangle rule. The compound as druglike has a higher chance of success and might can clear clinical trials with fewer side effects. The compound also revealed zero alert for PAINS (pan-assay interference compound), meaning that the compound is specific in binding to one biological target [83]. The compound also showed higher oral absorption and has high gastrointestinal absorption thus reaching a good concentration at the target site. The plasma protein binding value of the compound is 64.47% while the volume distribution score is 0.597. The compound is also inhibitor of CYP1A2, CYP2C19, CYP2C9, CYP2D6, and CYP3A4 receptors. The compound also serves as a substrate for different receptors to exclude it from the cell as xenobiotics. The compound was also found as non-acute toxic and non-mutagenic.

## Conclusion

The present study found Riddelline among the *T*. *articulate* phytochemicals as the highest affinity binder to antibacterial targets particularly the KdsA enzyme. The binding energy value of this compound was higher for anti-bacterial targets, anti-cancer and anti-viral targets compared to other compounds. As the docking studies give static intermolecular conformation and interactions pattern, the dynamics or physical movements in cellular environment was simulated and found the Riddelline-KdsA complex as stable with no major structure deviations reported. This confirms the compound long term stable binding with the enzyme’s active region. Further, intermolecular interactions were dominated by van der Waals forces, and less contribution was observed from electrostatic energy. The compound also shows good druglike properties, higher oral absorption and less toxicity. These findings altogether suggest the compounds as potent lead for additional structure optimization to accomplish higher bioactivity, oral bio-absorption and least mutagenicity and toxicity. The compound also needs to be tested in enzyme inhibition assay to evaluate its inhibition potential.

## Supporting information

S1 Table Phytochemicals detected from methanolic extract of TA by LC-MS. (DOCX)

S1 File Details ADMET properties of Riddellin compound. (PDF)
