# Supplementary material for: Riddelline from Tamarix articulate as a potential anti-bacterial lead compound for novel antibiotics discovery: A comprehensive computational and toxicological studies
Source: PLoS One. 2024 Nov 14;19(11):e0310319. doi: 10.1371/journal.pone.0310319 (PMC11563397; doi:10.1371/journal.pone.0310319)
Supplement: S1 Table — (DOCX) [file pone.0310319.s001.docx]

**S-Table 1.** Phytochemicals detected from methanolic extract of TA by LC-MS

| S. No | Compound |
| --- | --- |
| 1 | 3-Methylbutanoic acid;2-Methylbutyrate |
| 2 | Pyruvate oxime |
| 3 | Choline |
| 4 | Aromatic aldehyde; D-Glycerate |
| 5 | Bromoethane |
| 6 | Succinic acid |
| 7 | Gamma-Coniceine |
| 8 | 5-Amino-4-imidazole carboxylate;1-Methyl-4-nitroimidazole |
| 9 | L-Pipecolate |
| 10 | Itaconate;(E)-Glutaconate |
| 11 | (R)-2-Hydroxyisocaproate;6-Hydroxyhexanoic acid |
| 12 | L-Aspartate |
| 13 | p-Cymene |
| 14 | 2-Phenylacetamide |
| 15 | Hypoxanthine; Threonate |
| 16 | 4-Hydroxyphenylethanol; Styrene-cis-2,3-dihydrodiol |
| 17 | 4-Nitrophenol;2-Nitrophenol;3-Nitrophenol;3-Hydroxypicolinic acid |
| 18 | Hypoglycin; Arecaidine |
| 19 | trans-4-Hydroxycyclohexanecarboxylate |
| 20 | alpha-Ketoglutarate |
| 21 | Spermidine |
| 22 | Flupropanate |
| 23 | L-Lysine |
| 24 | D-Arabinono-1,4-lactone |
| 25 | Tolylacetate |
| 26 | Indole-3-acetaldehyde |
| 27 | 2-Oxoadipate |
| 28 | L-Carnitine |
| 29 | Methyl cinnamate; Safrole |
| 30 | Jasmone |
| 31 | Glycyl-leucine |
| 32 | Robinobiose |
| 33 | N-Formimino-L-glutamate |
| 34 | 3-Chloro-cis,cis-muconate |
| 35 | 4-Hydroxy-4-methylglutamate |
| 36 | L-Cladinose; Metaldehyde |
| 37 | D-Glucose |
| 38 | Glycyl-leucine |
| 39 | Carpacin;Myristicin |
| 40 | Linalyl acetate;alpha-Terpinyl acetate |
| 41 | L-Mimosine |
| 42 | beta-Caryophyllene |
| 43 | Chloroneb |
| 44 | 1,4-Dimethylphenanthrene |
| 45 | Fraxetin |
| 46 | Ammodendrine |
| 47 | Sedoheptulose |
| 48 | N-Acetylserotonin |
| 49 | Tigloidine |
| 50 | Aspidinol |
| 51 | Ametryn |
| 52 | **Resveratrol** |
| 53 | N-Acetyl-L-2-amino-6-oxopimelate |
| 54 | Alantolactone |
| 55 | (1R,6R)-6-Hydroxy-2-succinylcyclohexa-2,4-diene-1-carboxylate |
| 56 | L-Fuculose 1-phosphate |
| 57 | Anagyrine |
| 58 | Pyridoxal phosphate |
| 59 | 6-Hydroxymelatonin |
| 60 | Fenapanil |
| 61 | **Solenopsin A** |
| 62 | Apigeninidin |
| 63 | Chanoclavine-I |
| 64 | **Oxymatrine** |
| 65 | Brevicolline |
| 66 | Acetochlor |
| 67 | (5-L-Glutamyl)-L-glutamine |
| 68 | Biotin sulfone |
| 69 | O6-Methyl-2'-deoxyguanosine |
| 70 | Ostruthin |
| 71 | 2,3,9,10-Tetrahydroxyberbine |
| 72 | Tolclofos-methyl |
| 73 | 2-(3,5-Dichlorophenylcarbamoyl)-1,2-dimethylcyclopropane-1-carboxylic acid |
| 74 | Leucocyanidin;Gallocatechin |
| 75 | Leucocyanidin; Gallocatechin; Epigallocatechin |
| 76 | Tebuconazole |
| 77 | Diflubenzuron; Edifenphos |
| 78 | Nafenopin |
| 79 | Inosine-5'-carboxylate |
| 80 | 2,4,6-Triphenyl-1-hexene |
| 81 | Icosanoic acid |
| 82 | Isazofos |
| 83 | Dihydropteroate; Pyraclonil |
| 84 | **Dronabinol; Cannabidiol; Cannabichromene** |
| 85 | Fenbendazole S-oxide |
| 86 | Belladine |
| 87 | Phosmet |
| 88 | Lecanoric acid |
| 89 | Metconazole |
| 90 | Mugineic acid |
| 91 | beta-Citryl-L-glutamate |
| 92 | Digallate |
| 93 | Stylopine |
| 94 | Trichloroethanol glucuronide |
| 95 | Affinine |
| 96 | Sterigmatocystin |
| 97 | Monocrotaline |
| 98 | Fenuron |
| 99 | 12alpha-Methylpregna-4,9(11)-diene-3,20-dione |
| 100 | Fluazifop |
| 101 | Sethoxydim |
| 102 | N, N-Dimethylsphing-4-enine |
| 103 | Fluorodifen |
| 104 | 1,2-Dehydroreticuline |
| 105 | Lithospermoside |
| 106 | 3,7-Di-O-methylquercetin; Cirsiliol; Tricin |
| 107 | Carnosol |
| 108 | Trifluralin;Befuraline |
| 109 | **Dicumarol** |
| 110 | Catharanthine; Tabersonine |
| 111 | (13Z,16Z)-Docosadienoic acid |
| 112 | Azafenidin |
| 113 | 1,2,3,7,8-Pentachlorodibenzofuran |
| 114 | Glyceollin I; Glyceollin II |
| 115 | Diclofop methyl |
| 116 | Bifenox |
| 117 | Protodeoxyviolaceinic acid |
| 118 | Thiamine monophosphate |
| 119 | Gibberellin A53 |
| 120 | Riddelline |
| 121 | Petunidin |
| 122 | Palmatine |
| 123 | Vincamine; Yohimbine; Stemmadenine; |
| 124 | 22-Oxodocosanoate |
| 125 | S-Adenosylmethioninamine |
| 126 | Gentiopicrin |
| 127 | Arachidonyltrifluoromethane |
| 128 | Triflumuron |
| 129 | Isopenicillin N |
| 130 | Chlorthiophos |
| 131 | Chelirubine; Catalpol |
| 132 | Flufenacet |
| 133 | Xanthosine 5'-phosphate |
| 134 | Gibberellin A8 |
| 135 | Salicin 6-phosphate |
| 136 | 16-Methoxytabersonine; Hirsuteine |
| 137 | Anilofos |
| 137 | S-(2-Chloroethyl) glutathione |
| 138 | Bursehernin |
| 139 | 2-O-Caffeoylglucarate |
| 140 | Biocytin |
| 141 | Secologanate |
| 142 | Portulacaxanthin II;Swertiamarin;Geniposidic acid;Gardoside |
| 143 | **Ailanthone** |
| 144 | 9-cis-10'-Apo-beta-carotenal |
| 145 | 7-Methylguanosine 5'-phosphate |
| 146 | 2-Methoxyestradiol-17beta 3-sulfate |
| 147 | (8Z,11Z,14Z,17Z,20Z,23Z)-Hexacosahexaenoic acid |
| 148 | Sulfentrazone |
| 149 | 1-O-Sinapoyl-beta-D-glucose |
| 150 | Visnadin |
| 151 | 5-Phospho-alpha-D-ribose 1-diphosphate |
| 152 | Macarpine |
| 153 | Rotenone; Deguelin |
| 154 | Ximenic acid |
| 155 | delta-Tocotrienol |
| 156 | Aureothin |
| 157 | S-Adenosyl-4-methylthio-2-oxobutanoate |
| 158 | Ophiobolin A |
| 159 | Prednisolone acetate; Citreoviridin |
| 160 | Asperlicin C |
| 161 | Abyssinone V |
| 162 | Linustatin |
| 163 | Imibenconazole |
| 164 | Nicosulfuron; Spirodiclofen |
| 165 | 1-Palmitoylglycerol 3-phosphate |
| 166 | 5-Dehydroavenasterol; Avenastenone |
| 167 | Cyclopamine |
| 168 | Imazosulfuron; Pyraflufen-ethyl |
| 169 | Pyraflufen-ethyl |
| 170 | Flusulfamide |
| 171 | (7R)-7-(5-Carboxy-5-oxopentanoyl) aminocephalosporinate |
| 172 | Yamogenin |
| 173 | Daidzin; Frangulin A; Puerarin |
| 174 | Casimiroedine |
| 175 | 5-Fluorouridine diphosphate |
| 176 | Plicatic acid |
| 177 | alpha-Phocaecholic acid |
| 178 | Abscisic acid glucose ester |
| 179 | Adenosine 3',5'-bisphosphate |
| 180 | Vicianin |
| 181 | epsilon-Rhodomycinone; Quizalofop-P-tefuryl |
| 182 | Nummularine F |
| 183 | Imperialine |
| 184 | Hentriacontane |
| 185 | Lunarine |
| 186 | Hallactone B |
| 187 | Soyasapogenol C; Oleanolic aldehyde |
| 189 | Mallotochromene |
| 190 | Cephamycin C; Glycitin |
| 191 | Cinchonain 1a |
| 192 | Phylloquinol |
| 193 | epsilon-Viniferin |
| 194 | Amygdalin |
| 195 | 5-Methyltetrahydrofolate |
| 196 | Paeonolide |
| 197 | Sophoranone |
| 198 | Protopanaxadiol |
| 199 | **Quercetin 3,3'-bissulfate;Quercetin 3,4'-bissulfate** |
| 200 | Luteolin 7-O-glucuronide |
| 201 | Plicamine |
| 202 | Agnuside |
| 203 | Cephaeline |
| 204 | Gypsogenin |
| 205 | alpha-Tocopherol acetate |
| 206 | CMP-N-trimethyl-2-aminoethylphosphonate |
| 207 | Stigmatellin |
| 208 | 5-Fluorodeoxyuridine triphosphate |
| 209 | Rutaevin;Haplodimerine;Nafenopin glucuronide |
| 210 | Asiatic acid |
| 211 | CDP-choline |
| 212 | dATP |
| 213 | 5'-Methoxyhydnocarpin-D |
| 214 | Absinthin |
| 215 | **Rottlerin** |
| 216 | Cucurbitacin D |
| 217 | Retinyl palmitate |
| 218 | Oleuropein |
| 219 | Zeinoxanthin;beta-Cryptoxanthin |
| 220 | Santiaguine |
| 221 | Verbascoside |
| 222 | Resiniferatoxin |
